# Impacts of Low-cost Robotic Pets for Older Adults and People With Dementia: Scoping Review

**DOI:** 10.2196/25340

**Published:** 2021-02-12

**Authors:** Wei Qi Koh, Faith Xin Hui Ang, Dympna Casey

**Affiliations:** 1 National University of Ireland Galway Galway Ireland; 2 Tan Tock Seng Hospital Singapore Singapore

**Keywords:** social robot, assistive technology, robotic animals, pet robots, older adults, dementia, low-cost robot, psychosocial intervention, intervention, robot, review, intervention

## Abstract

**Background:**

Older adults and people with dementia are particularly vulnerable to social isolation. Social robots, including robotic pets, are promising technological interventions that can benefit the psychosocial health of older adults and people with dementia. However, issues such as high costs can lead to a lack of equal access and concerns about infection control. Although there are previous reviews on the use of robotic pets for older adults and people with dementia, none have included or had a focus on low-cost and familiarly and realistically designed pet robots.

**Objective:**

The aim of this review is to synthesize evidence on the delivery and impact of low-cost, familiarly and realistically designed interactive robotic pets for older adults and people with dementia.

**Methods:**

The Arksey and O’Malley framework was used to guide this review. First, the research question was identified. Second, searches were conducted on five electronic databases and Google Scholar. Studies were selected using a two-phase screening process, where two reviewers independently screened and extracted data using a standardized data extraction form. Finally, the results were discussed, categorized, and presented narratively.

**Results:**

A total of 9 studies were included in the review. Positive impacts related to several psychosocial domains, including mood and affect, communication and social interaction, companionship, and other well-being outcomes. Issues and concerns associated with its use included misperceptions of the robotic pets as a live animal, ethical issues of attachment, negative reactions by users, and other pragmatic concerns such as hygiene and cost.

**Conclusions:**

Overall, the findings resonate with previous studies that investigated the effectiveness of other social robots, demonstrating the promise of these low-cost robotic pets in addressing the psychosocial needs of older adults and people with dementia. The affordability of these robotic pets appeared to influence the practicalities of real-world use, such as intervention delivery and infection control, which are especially relevant in light of COVID-19. Moving forward, studies should also consider comparing the effects of these low-cost robots with other robotic pets.

## Introduction

The incidence of dementia increases with age [1], as such, it is one of the biggest challenges associated with a rapidly ageing population worldwide [2]. Older adults and people with dementia are especially susceptible to social isolation and loneliness [3-5], which can further dispose them to other morbidities such as decreased resistance to infection [6], depression, and further decline in cognitive functions [7]. This issue is especially pertinent with the ongoing COVID-19 pandemic [8], where older adults are largely confined within the home or residential care settings. Therefore, there is a need for innovative solutions to address the psychosocial needs of this population.

With technological advancements, promising innovations such as social robots have been developed to render emotional support and companionship [9,10]. A social robot may be defined as “an autonomous or semi-autonomous robot that interacts and communicates with humans by following the behavioural norms expected by the people with whom the robot is intended to interact” [11]. Robotic pets are a type of social robot with the appearance and behaviors of pets or companion animals [12]. A recent systematic review was conducted to understand the experiences and effects of older adults’ interactions with robotic pets in residential care facilities [13]. A total of five types of pet robots were identified across 19 studies, including 2 robotic cats (NeCoRo and JustoCat), a dog-like robot (AIBO), a robotic teddy bear (CuDDler), and a seal-like robot (Paro). The review showed that these robotic pets had positive benefits on psychosocial domains such as reduced agitation, reduced loneliness, and improved quality of life. These findings are congruent with another recent systematic review that similarly found the positive psychosocial benefits of using social robots in improving engagement and interaction, and reducing loneliness for older adults and people with dementia [14].

Despite positive benefits, there are important issues that may impede the uptake of robotic pets beyond the research setting. Some authors have argued that researchers appear to have a selection bias toward using Paro [15], which is one of the most widely deployed social robots in research to date [16]. Paro was designed to resemble an unfamiliar animal to improve its acceptability to users, based on the premise that users would have less preconceptions or expectations of it as compared to a familiar animal [17]. Nevertheless, it is worth considering that design preferences are unique and may differ across individuals. For instance, a recent study [15] showed that roboticists chose Paro as their preferred design while none of the older adults chose it. Instead, most chose the Joy for All (JfA) robotic cat and dog as their preferred designs and reported stronger preferences for familiarly designed robotic pets over unfamiliar ones such as Paro. Nonrealistic robotic pets such as Pleo, a robotic dinosaur, were also not preferred by older adults. Such preferences have been demonstrated in other studies [18-20], where older adults and people with dementia reported a preference for more familiar and realistic robotic pets such as a cat or dog. Hence, there is value in exploring the impacts of pet robots that are both familiarly and realistically designed.

Another impediment to the uptake of robotic pets relates to cost, which has been widely cited as a pragmatic concern by multiple key stakeholders including older end users [21], family members [18], organizations, and researchers [22-24]. For instance, each unit of the Justocat costs about US $1350, an AIBO dog costs US $3000, and a Paro costs approximately US $6000. Cost and affordability can therefore influence equal access to such innovations by older adults and people with dementia [25]. Furthermore, the high cost of social robots may make it difficult for older adults to own individual social robots. Instead, they are often shared among users [13]. This then raises concerns about hygiene and infection control [22,26]. In light of COVID-19, the issue of infection control is especially pertinent, as shared use may increase the risk of transmission of infections between users [27,28]. In fact, the shared use of robotic pets within care settings has recently been advised against [29]. Therefore, there is value in exploring lower cost alternatives.

**Figure 1:**
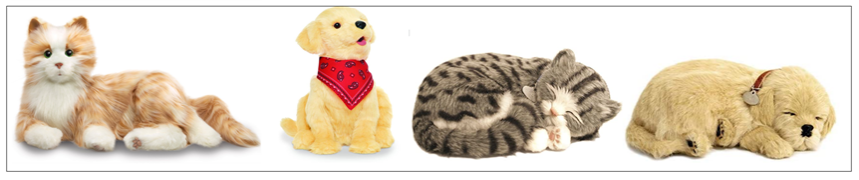
Low-cost, familiarly designed robotic pets and toys. Left to right: Joy for All cat, Joy for All dog, Perfect Petzzz cat, Perfect Petzzz dog.

Bradwell et al [15] identified several commercially available robotic pets. Among them, those that are low-cost and are realistically and familiarly designed include the Perfect Petzzz pets as well as the JfA robotic pets [15] (Figure 1). The Perfect Petzzz cats and dogs costs between US $15-$35; however, they are noninteractive in nature, and they may be considered as toys rather than social robots [30]. On the other hand, the JfA robotic cat and dog have interactive features and contain touch- and light–activated sensors to enable autonomous responses through vocalizations and movements for the purpose of social interaction. Although they are objectively less technologically advanced and cannot be programmed, older adults perceived them to be highly interactive as compared to another more technologically advanced robot [31]. As each unit of the JfA robotic pet costs between US $110-$130 (as of November 2020) [32], they are substantially more affordable. Furthermore, a cost-effectiveness study, which evaluated the use of a robotic pet with advanced touch capacities for people with dementia in long-term care settings, showed that a plush toy alternative offered marginally greater value for money [33]. Therefore, even though the JfA robotic pets have less technological features, they may be promising as a low-cost solution to address the psychosocial needs of older adults and people with dementia.

Although there has been previous reviews on the use of robotic pets for older adults [13], none have included or had a focus on low-cost, familiar, and realistically designed robotic pets. To the best of our knowledge, the JfA robotic pets are the only commercially available robotic pets that meet all three criteria as previously established. As such, the aim of this scoping review is to synthesize evidence on the delivery and impact of familiarly and realistically designed low-cost interactive robotic pets (ie, the JfA robotic cat and dog) for older adults and people with dementia. A scoping review methodology was chosen, as it is well suited to explore the breadth and depth of literature in this field [34].

## Methods

This scoping review follows the methodological framework proposed by Arksey and O’Malley [35], which includes five stages. The stages of conducting the review and analysis were as follows.

### Stage 1: Identification of the Research Question

The research question for this scoping review is “What is known about the impacts of low-cost, familiarly and realistically designed interactive robotic pets (i.e., the JfA robotic dog and cat) for older adults and people with dementia?”

### Stage 2: Identification of Relevant Studies

Published articles and grey literature were identified and searched in the following electronic databases: CINAHL, Web of Science, Scopus, MEDLINE via Ovid, and PsycINFO via Ovid. All relevant literature that were written in English, regardless of methodological quality, were included. Since the JfA robotic pets were only developed in 2016, only studies published after 2016 were included. The search strategies were developed in consultation with a research librarian based on the *Population, Concept, and Context* (*PCC*) framework that is recommended by the Joanna Briggs Institute for scoping reviews (Textbox 1). The full search strategy can be found in Multimedia Appendix 1. To cover the breadth of available literature and to ensure that the search was comprehensive, searches were also conducted on Google Scholar and through forward and backward citation tracing. The search was initially conducted in May 2020. To maximize the currency of this review [36], an update of the search was conducted in September 2020.

**The Population, Concept, and Context framework.**

**Population**

Older adults (60 years and older) and people with dementia

**Concept**

Interventions using low-cost and realistically and familiarly designed robotic pets (ie, the Joy for All robotic cat and dog)

**Context**

No limits applied to the study context (eg, participants’ homes, care settings)

### Stage 3: Selection of Studies

**Figure 2:**
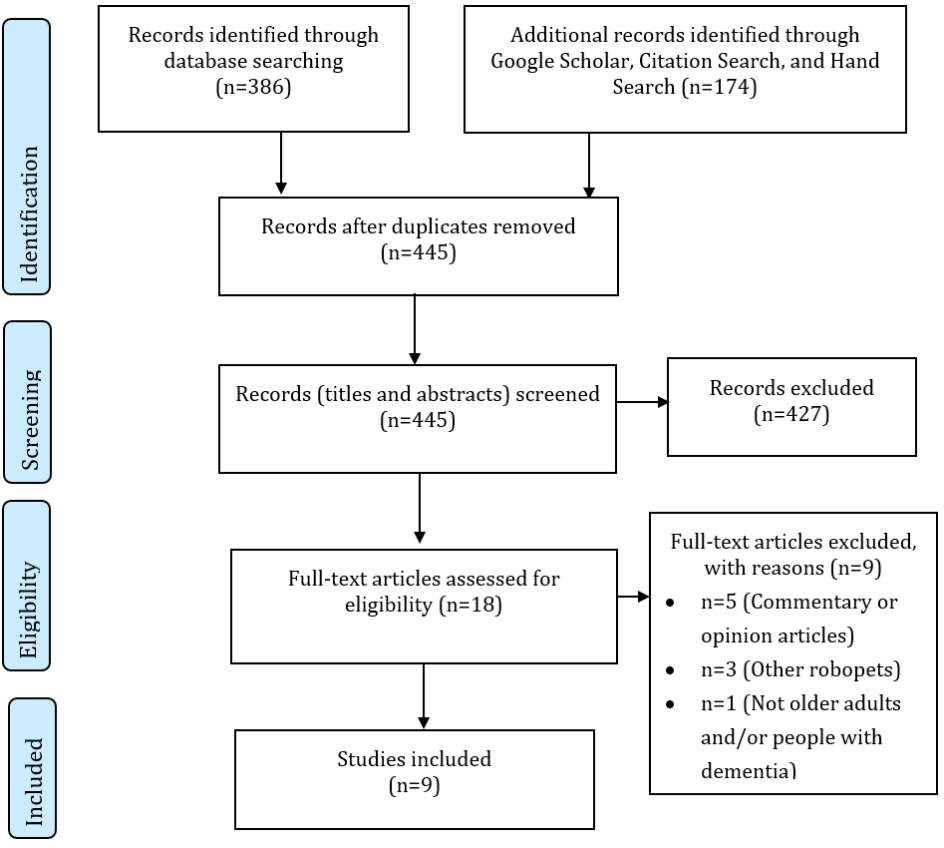
PRISMA (Preferred Reporting Items for Systematic Reviews and Meta-Analyses) flowchart.

The selection of studies followed a two-stage screening process. Two independent reviewers (authors WQK and FXHA) were involved in the screening process. Any nonconsensus or discrepancies were discussed and resolved among both reviewers and with author DC, as necessary. First, the titles and abstracts of identified articles were independently screened. We anticipated that information regarding the specific type of robotic pet (ie, the JfA robotic cat and dog) may not be mentioned in the title or abstract of publications and may only be available in the body of the text. Therefore, all studies were included if they met the following inclusion criteria based on the PCC framework: had any type of primary study; used a robotic cat or dog as an intervention; included older adults 60 years or older, or people with dementia; and were published in the English language. The exclusion criteria included if they were noninterventional studies such as expert opinion and commentaries, used any other robotic pets such as Pleo or AIBO, did not include older adults (ie, younger than 60 years), and were published in languages other than English. If these criteria were unclear in the title and abstract screening, they were included for full-text screening. Second, the full texts of included articles were reviewed. Studies that employed the JfA robotic pets were included, and studies using any other robotic pets such as the Justocat and NeCoRo cat were excluded. Any disparities were discussed and resolved. A bibliographic reference management tool, EndNote, was used to ensure that all articles were systematically accounted for. The search strategy was recorded using a PRISMA (Preferred Reporting Items for Systematic Reviews and Meta-Analyses) flowchart (Figure 2).

### Stages 4 and 5: Charting the Data and Summarizing and Reporting the Results

A standardized data extraction form was created using Excel (Microsoft Corporation). The data that were extracted included authors, country of the study, research design, research setting, participants’ demographics, sample size, intervention delivery, positive impacts, and negative impacts. Authors of included studies were contacted as necessary to attain additional information. Both reviewers (WQK and FXHA) charted the data independently before making comparisons afterward. Both reviewers discussed to collate the extracted data into categories and refined them to develop the final themes. The PRISMA-ScR (Preferred Reporting Items for Systematic reviews and Meta-Analyses extension for Scoping Reviews) checklist (Multimedia Appendix 2) was used to guide the reporting of the results [37].

## Results

A total of 9 publications were included in the final review.

### Quality Appraisal

Although quality appraisal is not necessitated for scoping reviews, it has been recommended to evaluate the methodological integrity of included articles [38]. Two reviewers (WQK and FXHA) independently appraised the quality of the included studies before meeting to discuss any discrepancies, which were resolved through discussion and a consensus was reached.

Qualitative studies and the qualitative strand of the mixed method study were appraised using the Critical Appraisal Skills Program qualitative checklist [39]. The research aims and rationale of all studies (n=7) were clearly stated. With the exception of 1 study [40], most studies confirmed that ethical approval was obtained from a relevant research ethics committee. Most had appropriate research designs (n=4) [40-43] and recruitment strategies (n=5) [40,41,43-45]. However, the data collection and analysis methods were not clearly described in 4 studies [42,46,47]. These factors subject the studies to assessor bias and reporting biases [48]. Emails were sent to the authors to request for more information; however, no responses were received. Most studies (n=6) did not provide sufficient information to illustrate if the relationship between the researchers and participants were adequately considered [40-43,45,47].

The National Institutes of Health (NIH) quality assessment tool for pre-post studies [49] was used to appraise the quantitative study and quantitative strand of the mixed method study. The tool contains 12 questions to guide reviewers’ judgement of whether a study is of “good,” “fair,” or “poor” quality. The quality of these studies were rated as poor and fair, respectively. In the mixed method study by Marsilio et al [45], it was unclear whether all eligible participants were enrolled, which subjected it to selection bias. In addition, the intervention was not clearly described, suggesting the potential for information bias. The other study by Tkatch et al [50] had a significant attrition rate. Furthermore, both studies did not state whether assessors were blinded, which raised concerns about reporting biases [45,50].

Finally, the Authority, Accuracy, Coverage, Objectivity, Date, Significance (AACODS) checklist [51] for appraising grey literature was used to evaluate the quality of McBride et al’s [46] article. This article did not have a clearly stated aim or research design. An email was sent to the authors requesting more information, and an author clarified that the study was *unstructured*, and there was no additional information beyond what was presented in the article. Hence, this article was rated to be of poor quality. The full quality appraisal tables can be found in Multimedia Appendix 3.

Overall, the quality of reporting in the included studies varied from poor to good, with most classified to be of poor to fair quality. Nevertheless, all studies were included in this scoping review, as the intention of this review is to identify the breadth of literature in this topic (Table 1).

**Table 1:** Characteristics of included studies.

Author	Aim	Robotic pet and cost	Method	Setting	Participants	Outcome measures
McBride et al 2017 [46]	Not clearly stated, appears to have explored impacts of low-cost interactive robotic pets for older residents	JfA^a^ cat and dog (US $99-$119 per unit)	Not clearly stated	Nursing home	Older adults living in residential care (n=33)	Clinical observation
Picking and Pike 2017 [47]	To explore the potential of an affordable robot, with a view to making a realistic difference in quality of life for people with dementia and their carers	JfA cat (<£100 [US $136.90] per unit)	Qualitative (multiple case study)	Own homes	Older people with dementia (n=3)	In-depth interviews with participants and carers, where they are encouraged to tell their story using aids such as photographs
Marsilio et al 2018 [45]	To determine whether introducing a robotic companion cat into a long-term care facility may improve affect and increase participation for residents with dementia; determine potential benefits for caregiver roles and relationships with individuals with dementia	JfA cat (no info on cost)	Mixed method	Nursing home	Long-term care facility residents with dementia (required assistance for some or all activities of daily living; n=11)	Agitation, using the Cohen-Mansfield Agitation Inventory Physiological measures (heart rate and oxygen saturation) Changes in the use of psychotropic and pain medications (review of the medication dispensing record) Clinical observations and staff report of participants’ behavior Questionnaire post study to evaluate staff perceptions of the effects of the robot on participants
Pike et al 2018 [42]	To explore the effects of a robot cat as companion robots for people living with dementia in their own homes	JfA cat (no info on cost)	Qualitative (multiple case study)	Own homes	Older people with dementia or early symptoms of dementia (n=6)	Interviews with people with dementia and their family, using photo elicitation when a photograph was available
Brecher 2019 [40]	To describe a case study on the effectiveness of using a robotic cat to successfully assist in the treatment of a patient with terminal restlessness	JfA cat (<US $100 per unit)	Qualitative (case report)	Veteran Affairs community living center	Older person with dementia (n=1)	Clinical observation
Bradwell et al 2020 [41]	To report ecologically valid diary data from two supported living facilities for older people with dementia or learning difficulties	JfA cat and dog (~£100 [US $136.90] per unit)	Qualitative (descriptive qualitative)	Two supported living facilities	Older adults with dementia or learning disabilities (no info on number of participants)	Diary entry by two members of staff at each supported living facility, using event-based sampling (ie, observations are logged after each observation) over a period of 6 months
Pike et al 2020 [43]	To investigate the use of robotic companion robots for people with dementia living at home with family or carer support	JfA cat (£100 [US $136.90])	Qualitative (multiple case study)	Own homes	Older adults with dementia or early symptoms of dementia (n=6)	Multiple interviews with participants and their family: first interview 2 weeks after they receive the cat and second interview at 3 months
Hudson et al 2020 [44]	To explore the efficacy of robotic pets in alleviating loneliness for older adults	JfA cat and dog (US $109.99-$129.90 per unit)	Qualitative (descriptive qualitative)	Own homes	Community-dwelling older adults (n=20)	Individual in-depth interviews
Tkatch et al 2020 [50]	To determine the feasibility of an animatronic pet program and whether ownership of animatronic pets would decrease loneliness and improve well-being among lonely older adults	JfA cat and dog (US $109.99-$129.90 per unit)	Quantitative (cohort study)	Own homes	Community-dwelling older adults (n=216)	Quality of life, using the VR-12^b^ Loneliness, using the UCLA^c^ Loneliness scale Resilience, using the BRS^d^ Purpose in life, using the NIH^e^ Tuberculosis Meaning and Purpose Scale Age 18+ Optimism, using the LOT-R^f^

^a^JfA: Joy for All.

^b^VR-12: Veteran's RAND.

^c^UCLA: University of California, Los Angeles.

^d^BRS: Brief Resilience Scale.

^e^NIH: National Institutes of Health.

^f^LOT-R: Life Orientation Test-Revised.

### Participants and Study Settings

The sample sizes in 8 studies ranged from 1 to 216 and included a total of 296 participants. It was not possible to ascertain the sample size in 1 study [41]. Most studies (n=6) were conducted with older adults with dementia [40-43,45,47]. However, 1 study also included older people with learning disabilities [41]. Healthy older adults were the participants in 2 studies [44,50]. In the remaining study, participants were older residents in a nursing home. However, there was no information on their ages or diagnoses [46]. Studies were conducted in participants’ homes (n=5) and in long-term care settings (n=4).

### Intervention Delivery

The majority (n=5) used the JfA robotic cat [40,42,43,45,47], while the others (n=4) employed both the robotic cat and dog [41,44,46,50]. Only 1 study offered participants’ their choice of robotic pet (ie, cat or dog) and reported no differences between the type of pet to the intervention outcomes [44]. The intervention duration ranged from 2 weeks to 6 months. The majority (n=9) delivered the robotic pet as a one-to-one intervention. Only 1 delivered the intervention both individually and communally [41]. Most (n=5) provided the robopet to participants on a full-time basis [42-44,50]. In 1 study, their use progressed from structured 1-2 hour sessions during the first 2-3 months to full-time use by the third month [41]. Finally, 2 studies reported intervention delivery on a weekly basis, between 1-3 times each week [41,46].

In most studies (n=7), minimal facilitation or instructions were provided by the researchers to guide intervention delivery with the robotic pets to allow their use to be scaffolded naturally [40-44,47,50]. Among studies that provided information about intervention delivery during the research, 3 reported facilitation by formal caregivers [41,45,46]. In 1 study, staff placed the robotic pet in the resident’s arm, talked about it, and then left the resident alone with it [45]. It was also made available during other times when residents asked for it or when the nurses were motivated to use the robotic pet with residents. Another study reported that, although the robotic pets were available in communal areas for unfacilitated interactions, structured group sessions with the robotic pets were also delivered by staff [41]. Finally, difficulties integrating the use of the robotic pets into nursing routines were reported in 1 study [46]. As such, nurses relied on therapeutic recreation staff to use them with nursing home residents [46].

### Positive Impacts of the Robotic Pets

The positive impacts included improved mood and affect, improved communication and interaction, companionship, and other well-being outcomes (Table 2).

**Table 2:** Positive impacts of the robotic pets.

Author (study setting)	Mood and affect	Communication and social interaction	Companionship	Well-being outcomes
McBride et al [46] (nursing home)	✓^a^	✓	—^b^	—
Picking and Pike [47] (participants’ homes)	✓	✓	—	—
Marsilio et al [45] (nursing home)	✓	✓	—	—
Pike et al [42] (participants’ homes)	—	✓	✓	—
Brecher [40] (nursing home)	✓	—	—	—
Bradwell et al [41] (assisted living)	✓	—	—	—
Pike et al [43] (participants’ homes)	✓	✓	✓	—
Hudson et al [44] (participants’ homes)	✓	✓	✓	—
Tkatch et al [50] (participants’ homes)	—	—	—	✓

^a^Observed in this study.

^b^Not observed in this study.

#### Improved Mood and Affect

Reduced agitation among older people with dementia was reported in 5 studies. Only 1 study used the Cohen-Mansfield Agitation Inventory and physiological indexes, and evaluated medication records to measure effects on agitation quantitatively [45]. Results showed statistically significant improvements in participants’ agitation scores and oxygen saturation. Nevertheless, there were no significant changes to participants’ heart rates. There were also no changes to the use of psychotropic or pain medications. Other studies reported their results based on observational data, where use of the robotic pets was reported to reduce aggression and disruptive behaviors [40,41,46]. The robotic pets were also found to be useful in de-escalating situations when people with dementia were agitated or anxious by providing calming effects [43,45-47]. Brecher [40] reported that a participant’s physical aggression almost completely resolved within 24 hours of interacting with the robotic pet. Similar effects were reported in other studies, where behavioral issues were described as having been reduced [45,46]. This calming effect was also reported by older people without cognitive impairments [44].

#### Communication and Social Interaction

The robotic pets were found to have positive impacts on participants’ communication and social interactions (n=8). When participants used the robopets in the presence of others, conversations and social interactions were facilitated [41-46]. In a study that was conducted to evaluate community-dwelling older adults’ experiences of using robotic pets, participants shared that their opportunities to connect with others was increased through sharing their pets in public spaces [44]. For people with dementia, the robopets provided a topic of conversation, which increased social interaction between participants and their care providers, family members, and other residents [41-43]. Furthermore, the robotic pets’ interactivity such as movements and sounds were observed to facilitate participants’ interaction with the pet or with others [41,43,45,46]. However, during unfacilitated robot interactions, some people with dementia were unaware that they needed to pet the cat to stimulate responses and reported concerns that their robopet had not interacted with them [45]. In such instances, staff had to prompt residents to touch the robot.

#### Companionship

People with dementia were reported to have developed companionship with their robotic pets [41,42,45,47] and in some instances had *formed a strong bond and attachment* with the robotic pets [41]. Only 1 study conducted a quantitative evaluation of loneliness with cognitively healthy older adults using the University of California, Los Angeles (UCLA) loneliness scale. Results showed a statistically significant decrease in older adults’ perception of subjective loneliness after 1 month of using the robotic pets [50]. This change was sustained after a second month of use. In the subsequent qualitative study, older adults shared similar sentiments that their perception of loneliness had reduced due to the presence of and interactions with the robopets [44]. This sense of presence was perceived to be comforting and enjoyable [43,44].

#### Other Well-being Outcomes

Quantitative measures of other outcomes were reported in 1 study [50]. In this study, there were no improvements to physical well-being of cognitively healthy older adults as recorded on the physical component of the Veteran’s RAND (VR-12). However, their mental well-being, resilience, and purpose in life, as measured on the mental component of VR-12, the Brief Resilience Scale, and the adapted version of the NIH Tuberculosis Meaning and Purpose scales, respectively, showed statistically significant improvements after 1-2 months of using the robotic pets. In a qualitative study that investigated the use of robotic cats for people with dementia living at home, interviews with family members revealed that the pet robot provided participants with a sense of purpose, which led to an overall improvement in well-being and function [43]. As a result, one of the participants in the study did not have to move to a residential care facility.

### Issues and Concerns Relating to Use of the Robotic Pets

Issues and concerns related to the use of the robotic pets included misperception and attachment, no impact or negative impacts, and practical issues.

#### Misperception and Attachment

Staff members in nursing homes reported that some people with dementia misperceived the robotic pets as live animals (n=2), which had implications on participants’ acceptance and interaction with the technology. In 1 study, some participants declined the pet robot as they did not want to be responsible for caring for the cat [45]. In another study, one participant requested for a cage and collar for the robotic pet and showed concerned about its care. Correspondingly, he became frustrated because of a perceived responsibility to care for the cat [46]. The issue of attachment to the robotic pets was also raised [41,45]. Some authors felt that attachment had the potential to cause emotional distress for users if a technical fault or breakdown were to occur [45]. In 1 study where participants shared the robotic pets in a group setting, some participants were reported to exhibit jealousy of others using the robot, as they were hesitant to share the robotic pets with others [41].

#### No Impact or Negative Impacts

Some participants with dementia declined or had no interactions with the robotic pets and reported negative preferences (ie, dislikes) toward animals [42,43,45,47]. Some participants perceived the robots as “creepy” and rejected their use [41,43]. The interactivity of the robots was also raised as an issue. Vocalizations of the robopet (eg, meowing) were reported to cause anxiety in a participant with dementia who felt concerned about its well-being [43]. In such instances, family members turned the robot off. Similarly, another participant with dementia who had active psychosis was reported to feel disturbed by the robopet’s sounds [46]. Some movements of the robotic cat, such as rolling over, also caused distress in some people with dementia, as they perceived that the cat was falling down [43]. A few participants exhibited agitation toward the robotic pet, and some attempted to harm it [41,45]. In 1 study, staff attributed the participant’s negative response to a recent change in psychotropic medications [45].

#### Practical Issues

Practical issues, which included cost, hygiene, and infection control, were raised. Although the low-cost of this innovation was cited as a reason for some researchers’ choice of social robot for their studies [40,41,43,50], other researchers and care staff also raised concerns about their affordability [41,44,50]. The issue of hygiene and infection control, such as through shared use in care facilities, was also brought up by staff and researchers in 2 studies as potential challenges for longer-term use [41,46]. The authors of 1 study suggested that the robotic pets should be kept off residents’ lap during mealtimes to address the issue of hygiene and that purchasing individual robots for each resident might simplify the issue of infection control [46].

## Discussion

### Principal Findings

This is the first scoping review to identify and synthesize the evidence on the delivery and impact of low-cost, familiarly and realistically designed robotic pets for older adults and people with dementia. The majority of the included studies in this review were conducted in long-term care facilities and in participants’ homes, and most employed the JfA robotic cat.

Overall, the positive impacts of the JfA robotic pets related to several psychosocial domains. Positive impacts included improved mood and affect, communication, social interaction, and companionship; these benefits resonate with findings in reviews that investigated the effectiveness of other social robots and robotic pets for older adults and people with dementia [13,14,16]. However, the impacts on other domains, including loneliness, resilience, and purpose in life, were less investigated; in this review, only 1 study that focused on cognitively healthy older adults reported on such outcomes [50]. This corresponded with findings from a review paper that investigated the use of social robots for older people [52] and found that only 3 studies reported outcomes relating to loneliness among healthy older adults. Similar to studies using other robotic animals, the interactivity of the JfA robotic cat and dogs have been described to facilitate users’ communication and interaction with the pet and with other people. Paradoxically, the interactive features of the JfA robopets caused distress among a few participants with dementia. Such issues have been reported previously, where users were disturbed by sounds produced by another robotic pet [18,53-55]. Moving forward, there is a need for robot developers to consider the customizability of the robopets’ interactive features in accordance with users’ preferences.

The issue of affordability has been reported to impede the use of robotic pets in the real world [18,21,22,24]. The low-cost of the JfA robotic pets appeared to have an influence on intervention delivery and the conduct of research; with the exception of 1 study, all participants in this review received their own robotic pet for individual use. This is in contrast to findings from a systematic review, which found that higher-cost robotic pets have been shared among users and used more frequently in group settings [13]. The affordability of the JfA robotic pets was also cited by researchers as one of the influencing factors in the choice of robotic pet for their studies [40,41,43,50]. Cost appeared to have played a role in influencing the research method in one study, where individual robopets were provided to 216 participants to enable a statistical significant analysis of their impacts [50]. This strategy may be more challenging to implement with more expensive robots [16]. In addition, it is worth noting that there is a relatively sizeable body of anecdotal evidence, largely stemming from individuals’ reports of their experiences with this technology [56-59]. This might also be attributed to their affordability, which might have enabled more users to gain access to this technology as compared to other social robots that are more expensive. For example, although Paro is one of the most researched social robots, it has substantially less user-generated reports of its impacts. This could be because Paro is primary used in institutions [17], likely due to its cost, which renders it to be less accessible for individual users’ purchase. Individual ownership of the robotic pets may be viewed as a promising way to mitigate the pertinent issue of infection control, especially in light of the ongoing COVID-19 pandemic. A recent publication by Bradwell et al [60] reported that the acceptable levels of microbes on robopets, including one with antibacterial fur covering [17], exceeded an acceptable threshold after 20 minutes of use. Frequent and shared use of these robopets between different users can further increase the potential of infection transmission [27,28]. Hence, since the lower cost of the JfA robopets increases the affordability of individual purchases for each user, the corresponding risk of direct or indirect contact transmission of infections related to shared use may be ameliorated.

Issues related to use of the JfA robopets were identified. Like other interventions involving social robots, there were issues associated with use of this intervention. Some participants with dementia did not benefit from their use or demonstrated negative responses toward the robopets. For this population, the ethical challenge of deception also emerged [10], as some participants misperceived them as real animals or showed attachment toward them. These issues are not unique to the JfA robotic cat and dog, as they have been reported in other studies using other robotic pets [23,33,61]. The significance of these issues should not be discounted, as those who were more attached or misperceived the robopets belonged to a vulnerable population. However, from the standpoint of the capability approach, all humans, including people with disabilities, should be given the opportunity to achieve a threshold level of core capabilities to uphold the principle of social justice [62]. Therefore, in consideration that the pet robot may facilitate a user’s capacities that would be otherwise undermined, such as facilitating social interaction, this can be viewed as enabling technology with greater benefits than risks [63]. In addition, formal and informal caregivers should also explicitly consider upholding this principle, particularly when delivering the robotic cat. When introducing this technology to users, they should introduce it as a robotic pet and refrain from referring to it as a real animal [63]. The understanding of potential issues such as jealousy and attachment may also guide future implementation and inform future robot development to ensure robustness of the technology.

Users’ responses toward the JfA robopets appear to be related to their profile (ie, preference for or experience with animals). Participants who did not respond or had negative responses to the JfA robopets were reported to not like animals. This aligned with findings from other studies that highlighted that multilevel stakeholders including people with dementia [17], family members [18], and staff [22] who liked animals had positive perceptions and reactions to robotic pets. Therefore, before considering the use of the JfA robopets to address the psychosocial needs of older adults or people with dementia, care providers should consider users’ preferences for animals, as well as their preferred type of robotic animals, to maximize the appropriateness and meaningfulness of the intervention.

### Strengths and Limitations

There are a number of strengths underpinning this work. First, the methodological framework used throughout the scoping review process was transparent and rigorous. The screening and data extraction process involved two independent reviewers, which reduced the risk of reviewer bias or article selection bias. Both reviewers met at regular intervals and discussed and resolved all discrepancies. Second, this paper discusses the pragmatic aspects relating to intervention delivery and the conduct of research using the JfA robotic pets, which can serve as useful considerations for researchers or users who are keen to further explore the use of this technology. However, there are limitations of this review. Articles that were published in other languages were not searched or included in this review. As non-English studies were excluded from this review, relevant studies may have been missed.

### Conclusions

This scoping review has mapped out current evidence on the use of and impact of realistic and familiarly designed low-cost robotic pets for older adults and people with dementia. Our review contributes to the evidence base that is necessary for more widespread awareness about the potential utility of these low-cost robotic pets to address the psychosocial needs of older adults and people with dementia, as both the positive impacts and issues related to their use largely resonate with research conducted with several other robotic animals. The affordability of these robopets appear to have an influence on intervention delivery. They also appear to have the ability to uphold the distributive justice of innovation dissemination; these are especially relevant in light of the COVID-19 pandemic, where there is an increased emphasis on infection control and equal access. However, more rigorous effectiveness trials are required to confirm their positive impacts. Future studies should also consider comparing the intervention effects of the JfA robotic pets with other robotic pets. It is also important to ascertain the design preferences of older adults and people with dementia to facilitate the development of future user-centered interventions using robotic pets. 

## Appendix

Multimedia Appendix 1 Search strategy.

Multimedia Appendix 2 PRISMA-ScR (Preferred Reporting Items for Systematic Reviews and Meta-Analyses extension for Scoping Reviews) checklist.

Multimedia Appendix 3 Quality appraisal.

## Abbreviations

AACODS scale: Authority, Accuracy, Coverage, Objectivity, Date, Significance scale
ITN: Innovative Training Network
JfA: Joy for All
NIH: National Institutes of Health
PCC: Population, Concept, and Context
PRISMA: Preferred Reporting Items for Systematic Reviews and Meta-Analyses
PRISMA-ScR: Preferred Reporting Items for Systematic reviews and Meta-Analyses extension for Scoping Reviews
VR-12: Veteran’s RAND
UCLA loneliness scale: University of California, Los Angeles loneliness scale
