# Supplementary material for: Impacts of Low-cost Robotic Pets for Older Adults and People With Dementia: Scoping Review
Source: JMIR Rehabil Assist Technol. 2021 Feb 12;8(1):e25340. doi: 10.2196/25340 (PMC8082946; doi:10.2196/25340)
Supplement: Multimedia Appendix 1 [file rehab_v8i1e25340_app1.pdf]

Supplementary File 1: Search strategy

Medline via Ovid

1. Robotics/
2. Robo*.mp.
3. 1 or 2
4. Animal Assisted Therapy/
5. Pets/
6. companion.mp.
7. 4 or 5 or 6
8. 3 and 7
9. robo* cat.mp.
10. robo* dog.mp.
11. robo* anima*.mp. [mp=title, abstract, original title, name of substance word, subject heading word, floating sub-heading word, keyword heading word, organism supplementary concept word, protocol supplementary concept word, rare disease supplementary concept word, unique identifier, synonyms]
12. 8 or 9 or 10 or 11
13. Aged/
14. (age or elderly or senior citizen* or older adult).mp. [mp=title, abstract, original title, name of substance word, subject heading word, floating sub-heading word, keyword heading word, organism supplementary concept word, protocol supplementary concept word, rare disease supplementary concept word, unique identifier, synonyms]
15. Dementia/
16. dementia.mp.
17. 15 or 16
18. 12 and 17
19. limit 18 to yr="2016 -Current"

PsycINFO via Ovid

1. Robotics/
2. Robo*.mp.
3. 1 or 2
4. Animal Assisted Therapy/
5. Pets/
6. companion.mp.
7. 4 or 5 or 6
8. 3 and 7
9. robo* cat.mp.
10. robo* dog.mp.
11. robo* anima*.mp. [mp=title, abstract, original title, name of substance word, subject heading word, floating sub-heading word, keyword heading word, organism supplementary concept word, protocol supplementary concept word, rare disease supplementary concept word, unique identifier, synonyms]
12. 8 or 9 or 10 or 11
13. Aged/
14. (age or elderly or senior citizen* or older adult).mp. [mp=title, abstract, original title, name of substance word, subject heading word, floating sub-heading word, keyword heading word, organism supplementary concept word, protocol supplementary concept word, rare disease supplementary concept word, unique identifier, synonyms]
15. Dementia/
16. dementia.mp.
17. 15 or 16
18. 12 and 17
19. limit 18 to yr="2016 -Current"

CINAHL

1. (MH "Robotics")
2. robo*
3. (MH "Pet Therapy")
4. animal assisted therapy
5. companion
7. S1 OR S2
8. S3 OR S4
9. S7 OR S8
10. robo* cat
11. robo* dog
12. robo* anima*
13. S9 OR S10 OR S11 OR S12
14. (MH "Aged")
15. "older adults"
16. (MH "Dementia")
17. dementia
18. S14 OR S15 OR S16 OR S17
19. S13 AND S19

(Limiters - Published date 2016/01/01 -)

Web of Science Core Collection

1. TOPIC: (robo*)
2. TOPIC: ("pet therapy")
3. TOPIC: ("animal assist* therapy")
4. TOPIC: ("robo* cat")
5. TOPIC: ("robo* dog")
6. TOPIC: ("robo* animal")
7. TOPIC: ("social robot")
8.TOPIC: ("social assistive robot")
9. TOPIC: ("socially asisstive robot"
10. TOPIC: ("companion robot")
11. #3 OR #2
12. #11 AND #1
13. #12 OR #10 OR #9 OR #8 OR #7 OR #6 OR #5 OR #4
14. TOPIC: ("older people")
15. TOPIC: ("older adult*")
16. TOPIC: ("elder*")
17. TOPIC: (dementia)
18. #17 OR #16 OR #15 OR #14
19. #18 AND #13
20. #18 AND #13

Refined by: PUBLICATION YEARS: (2020 OR 2019 OR 2018 OR 2017 OR 2016)

Scopus

1. TITLE-ABS-KEY (robo*)
2. TITLE-ABS-KEY ("animal assist* therapy")
3. TITLE-ABS-KEY ("pet therapy")
4. TITLE-ABS-KEY ("robo* cat")
5. TITLE-ABS-KEY ("robo* dog")
6. TITLE-ABS-KEY ("robo* pet")
7. TITLE-ABS-KEY ("robo* animal")
8. TITLE-ABS-KEY ("social* assist* robot"
9. TITLE-ABS-KEY ("companion robot")
10. #2 OR #3
11. #1 AND #10
12. #4 OR #5 OR #6 OR #7 OR #8 OR #9 OR #11
13. TITLE-ABS-KEY ("older people")
14. TITLE-ABS-KEY ("older adult*")
15. TITLE-ABS-KEY ("elder*")
16. TITLE-ABS-KEY (dementia)
17. #13 OR #14 OR #15 OR #16
18. #12 AND #17

Publication limit 2016 – 2020
