# Supplementary material for: Impacts of Low-cost Robotic Pets for Older Adults and People With Dementia: Scoping Review
Source: JMIR Rehabil Assist Technol. 2021 Feb 12;8(1):e25340. doi: 10.2196/25340 (PMC8082946; doi:10.2196/25340)
Supplement: Multimedia Appendix 3 [file rehab_v8i1e25340_app3.pdf]

Supplementary File 3: Quality Appraisal

Critical Appraisal Skills Program (CASP) Qualitative Checklist

Question number	Marsilio et al, 2018	Picking and Pike, 2017	Pike et al, 2018	Brecher et al, 2019	Bradwell et al, 2020	Pike et al, 2020	Hudson et al, 2020
1	Yes	Yes	Yes	Yes	Yes	Yes	Yes
2	Yes	Yes	Yes	Yes	Yes	Yes	Yes
3	Can't tell	Can't tell	Yes	Yes	Yes	Yes	Can't tell
4	Yes	Can't tell	Can't tell	Yes	Yes	Yes	Yes
5	Can't tell	Can't tell	Can't tell	Can't tell	Yes	Yes	Yes
6	No	No	No	No	No	Can't tell	Yes
7	Yes	Can't tell	Can't tell	No	Yes	Yes	Yes
8	No	No	No	Can't tell	Yes	Yes	Yes
9	Can't tell	No	No	Yes	Yes	Yes	Yes

NIH Quality Assessment Tool for Before-After (Pre-Post) Studies With No Control Group

Question number	Marsilio et al, 2018	Tkatch et al, 2020
1	Yes	Yes
2	Yes	Yes
3	Yes	Yes
4	CD	Yes
5	No	Yes
6	No	CD
7	Yes	Yes
8	No	CD
9	CD	No
10	Yes	Yes
11	No	No
12	NA	NA
Quality rating	Poor	Fair

AACODS Checklist

AACOD questions	McBride et al, 2017
Author	No
Accuracy	No
Coverage	No
Objectivity	No
Date	Yes
Significance	Can't tell
